# Urosymphyseal fistulas and their radiographic features: A case report

**DOI:** 10.1016/j.radcr.2025.07.037

**Published:** 2025-08-13

**Authors:** Alex Shoung, Charlie Chia-Tsong Hsu, Nicholas McKay Parry

**Affiliations:** a Department of Medical Imaging, Gold Coast Hospital and Health Service, 1 Hospital Boulevard, Southport, Queensland 4215, Australia; b School of Medicine and Dentistry, Griffith University, Southport, Queensland, Australia; c Division of Neuroradiology, Department of Medical Imaging, Gold Coast University Hospital, Southport, Queensland, Australia

**Keywords:** Urosymphyseal fistula, Prostate cancer, Pelvic radiotherapy, Computed tomography, Magnetic resonance imaging

## Abstract

Urosymphyseal fistulas are a rare but serious complication of prostate cancer treatment, particularly in patients with a history of pelvic radiotherapy. Diagnosis is often delayed due to the nonspecific nature of presenting symptoms. We report the case of a 67-year-old man who developed a urosymphyseal fistula following radiotherapy and multiple endoscopic bladder interventions. This report highlights the characteristic radiographic features observed on computed tomography and magnetic resonance imaging. Increased awareness of urosymphyseal fistulas, combined with the use of targeted imaging modalities, is essential for prompt diagnosis and effective management.

## Introduction

Prostate cancer is one of the most prevalent malignancies in males [1]. Despite high 5-year survival rates for early-stage diagnoses, prostate cancer remains the leading cause of male malignancy-related deaths in more than quarter of countries [1,2]. Although treatment options for prostate cancer have advanced considerably, surgery and radiotherapy remain the cornerstone of management [3].

Uro-symphyseal fistulas (USFs) are a rare complication of prostate cancer treatment, most commonly arising following radiotherapy [4]. They involve an abnormal communication between the lower urinary tract and the pubic symphysis, resulting in significant morbidity and potentially leading to complications such as pubic bone osteomyelitis and urosepsis [4,5]. Symptoms are often nonspecific, with pain on mobilization being the most common [4]. Additional symptoms may include pelvic pain and lower urinary tract symptoms [6]. The nonspecific presentation and rarity of USFs often contribute to delays in diagnosis [6]. Moreover, due to the limited number of reported cases, a consensus diagnostic and treatment guidelines has yet to be established [4,6]. Increasing clinician awareness of USFs and their imaging findings is vital to minimize diagnostic delays and improve patient outcomes.

We present a case of a USF and highlight its radiographic features on computed tomography (CT) and magnetic resonance imaging (MRI).

## Case report

A 67-year-old man presented to the emergency department with progressive suprapubic and bilateral groin pain that severely limited his mobility. His medical history was notable for a robot-assisted laparoscopic prostatectomy performed 10 years earlier for Gleason 5+4 prostate cancer, followed by salvage external beam radiation therapy 8 years ago due to rising prostate-specific antigen levels. In the preceding months, he had multiple admissions for hematuria and clot retention, requiring three cystodiathermies.

On examination, there was erythema and induration of the right medial thigh, accompanied by pain on both active and passive movement. Laboratory investigations revealed leukocytosis (white cell count 14.1 × 10^9^/L), neutrophilia (12.31 × 10^9^/L), and an elevated C-reactive protein level of 155 mg/L.

A portal venous phase CT scan of the abdomen and pelvis demonstrated a thick-walled urinary bladder adherent to the pubic symphysis with extensive surrounding fat stranding. There was minor cortical erosion of the pubic symphysis with multiple locules of gas in the adjacent tissues. Inflammatory changes extended into both adductor compartments, and a rim-enhancing collection measuring 42 × 21 × 66 mm was identified within the right adductor longus muscle.

These findings raised suspicion for a USF, which was confirmed on a delayed-phase CT scan performed 90 minutes later. The patient was managed acutely with an indwelling urinary catheter and broad-spectrum intravenous antibiotics. The right adductor compartment collection was drained under ultrasound guidance.

Urine cultures grew *Pseudomonas aeruginosa*, while cultures from the adductor compartment collection revealed mixed enteric bacteria. An MRI of the pelvis with intravenous gadolinium contrast confirmed osteomyelitis of the pubic symphysis.

The patient subsequently underwent a supratrigonal cystectomy with ileal conduit formation and excision of the pubic symphysis. He received additional postoperative antibiotics and made a good recovery.

## Discussion

USFs are an uncommon but serious complication following prostate cancer treatment [4]. Existing literature identifies prior pelvic radiotherapy as the biggest factor for the development of USFs, with subsequent bladder outlet procedures compounding this risk [4]. Interestingly, salvage radiotherapy is considered a greater risk factor than primary radiotherapy, likely due to its higher association with urethral stricture formation and the need for additional procedures [4]. Radiotherapy increases the risk of USF formation by causing tissue ischemia and radiation-induced fibrosis, impairing healing and making surrounding structures more susceptible to infection [6]. USF development often begins with disruption of the urethral mucosa, allowing microorganisms to penetrate deeper tissues [6]. This may explain why bladder outlet interventions, through their associated trauma, can precipitate the formation of USFs [6].

Cross-sectional imaging, particularly CT and MRI, play a critical role in identifying USFs and their complications. CT is often the initial imaging modality due to its accessibility, and is able to identify abscess formation and features indicative of chronic osteomyelitis [6,7]. On CT, chronic osteomyelitis can be characterized by cortical thickening, sclerotic remodeling, and the presence of a chronic draining sinus tract [7]. A subsequent delayed-phase or cystogram CT may then aid in confirming the presence and delineating the extent of the fistula [6]. MRI is widely regarded in literature as the gold standard for diagnosing USFs, offering superior visualization of fluid collections, early detection of osteomyelitis, and identification of adjacent myositis [[4], [5], [6], [7], [8]]. Osteomyelitis typically appears as decreased signal intensity on T1-weighted images and increased signal intensity on T2-weighted images, due to bone marrow inflammation and subsequent oedema [7]. Bacterial pyomyositis demonstrates signal intensity on T1-weighted MRI that varies with the protein content of the fluid, ranging from similar intensity to mildly increased compared to normal muscle [9]. On T2-weighted MRI, the affected muscle usually shows increased signal intensity [9].

**Fig. 1:**
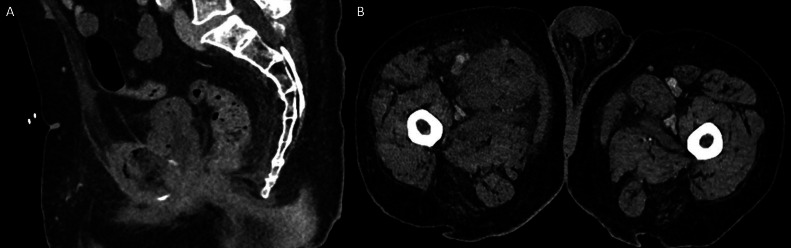
CT scan in the portal venous phase. (A) Sagittal slice showing a thick-walled urinary bladder adherent to the pubic symphysis with extensive surrounding fat stranding. Multiple gas locules are visible adjacent to the pubic symphysis. (B) Axial slice demonstrating inflammatory changes in the bilateral adductor compartments, more pronounced on the right, with a rim-enhancing collection within the right adductor longus and a smaller abscess in the adductor magnus.

**Fig. 2:**
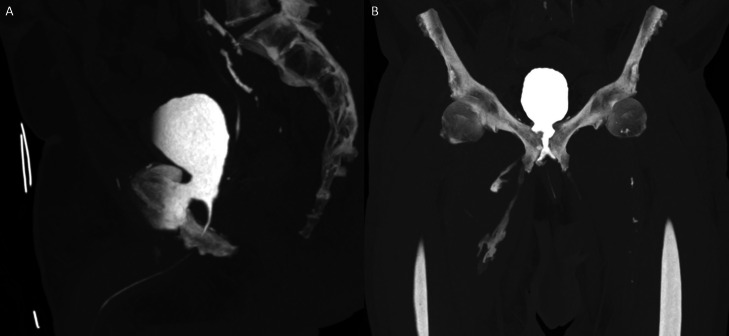
Delayed-phase CT scan performed 90 minutes later. (A) Sagittal slice demonstrating a direct fistulous tract between the anterior-inferior bladder wall and the pubic symphysis. (B) Coronal slice showing fistulous tracts extending into the bilateral adductor compartments.

**Fig. 3:**
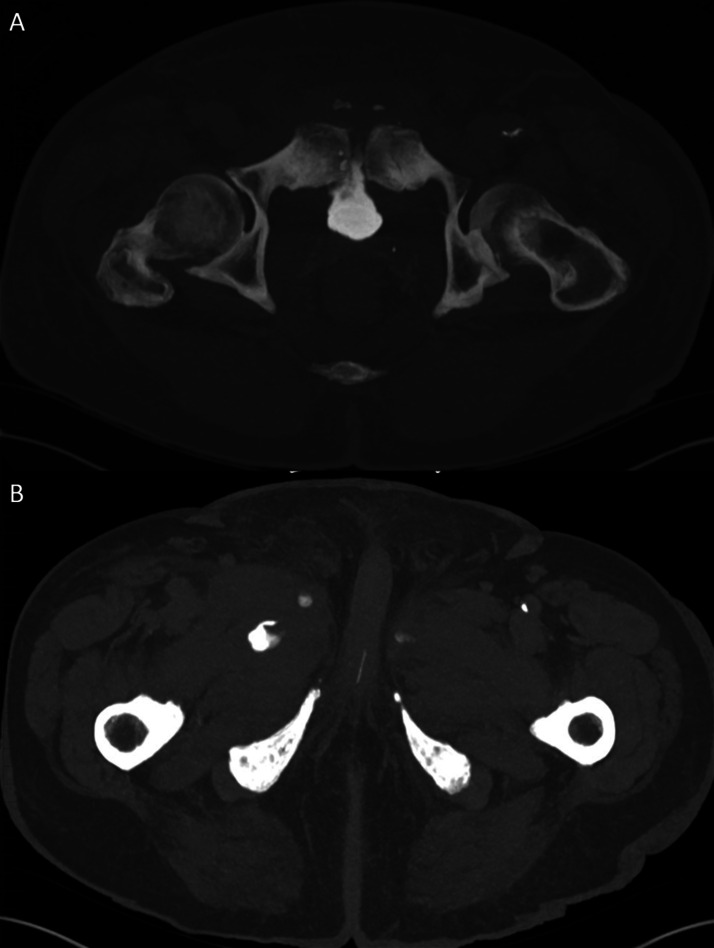
Delayed-phase CT demonstrating a direct fistulous tract between the bladder and pubic symphysis (A) and contrast leakage into the bilateral adductor compartments (B).

**Fig. 4:**
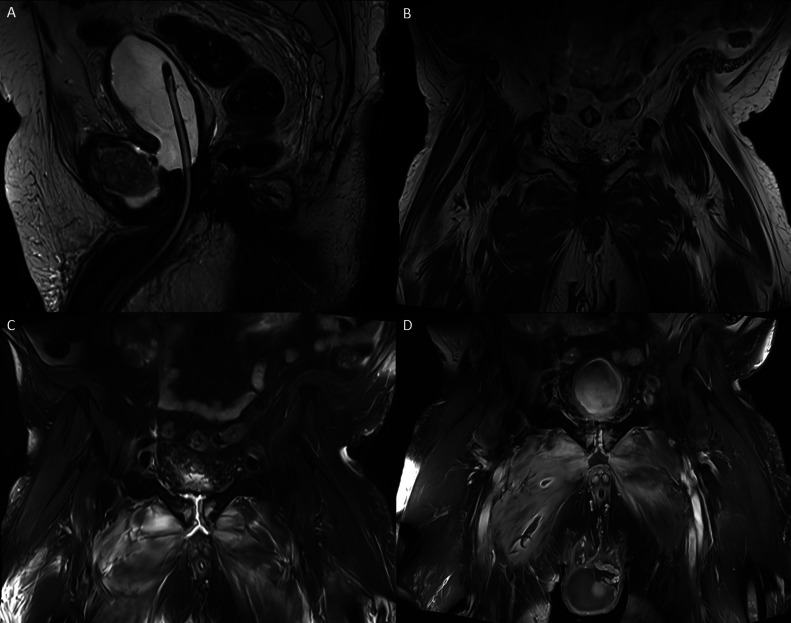
(A) Sagittal T2-weighted turbo spin echo MRI slice demonstrating direct communication between the anterior-inferior bladder and pubic symphysis. (B-D) Coronal MRI slices showing diastasis of the pubic symphysis with low signal intensity on T1 (B), high signal intensity on T2 (C), and enhancement on postcontrast T1 (D) of the medial pubic body margins. These findings are consistent with osteomyelitis. Additional features demonstrate bilateral abductor compartment myositis characterized by T1 isointensity (B) and T2 hyperintensity (C).

In our case, the initial portal venous phase CT revealed findings consistent with cystitis and abscess formation within the medial compartments of the thighs (Fig. 1). Delayed-phase imaging later demonstrated fistulous connections between the bladder wall, pubic symphysis, and bilateral adductor compartments (Fig. 2, Fig. 3). MRI confirmed osteomyelitis of the pubic symphysis, characterized by low signal intensity on T1-weighted images and high signal on T2-weighted sequences (Fig. 4). Additionally, features of pyomyositis were evident in the bilateral adductor muscles, appearing isointense on T1 and hyperintense on T2-weighted imaging (Fig. 4).

Conservative treatment of USFs with intravenous antibiotics and urinary catheterization is often ineffective, particularly in patients with a history of pelvic radiation [4]. While patients without prior radiation exposure generally experience more favorable outcomes, nearly all patients with previous radiation fail to respond to conservative management [4]. This high failure rate raises concerns about prolonged morbidity and the potential for adverse outcomes associated with initially trialing conservative management [4]. Cystectomy with urinary diversion is the most commonly employed surgical approach for managing USFs [4,6]. Although bladder-sparing techniques, such as primary repair with tissue flaps, may be considered in select cases, concerns remain regarding impaired healing and suboptimal outcomes in patients with a history of pelvic radiotherapy [4,6].

**Fig. 5:**
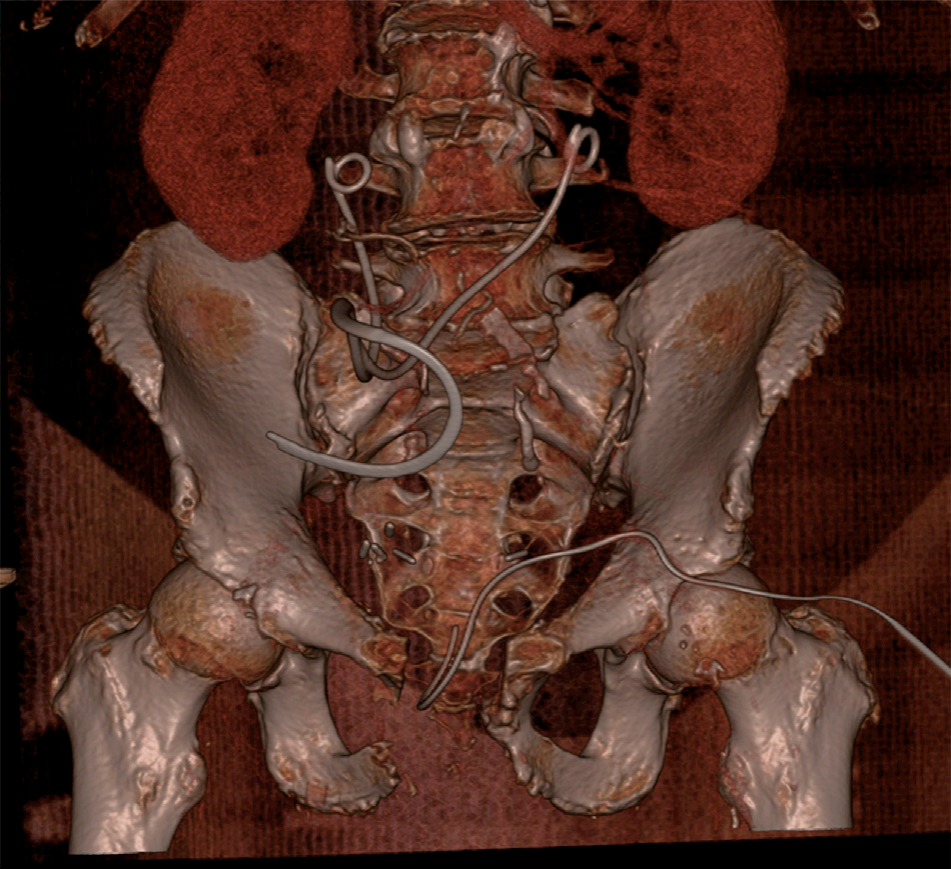
Postoperative reconstructed CT demonstrating bilateral ureteral stents exiting through the anterior abdominal wall via the ileal conduit, absence of the pubic symphysis, and the presence of a surgical drain.

USFs are a rare but serious complication of prostate cancer treatment, particularly in the context of prior pelvic radiotherapy. Diagnosis is challenging due to vague clinical symptoms, making appropriate imaging vital for timely identification of the fistula and its complications. Given the limited success of conservative management in radiated patients, surgical intervention, typically a cystectomy with urinary diversion, is often necessary (Fig. 5). Greater awareness of USFs and their radiologic features is essential for timely diagnosis and improved patient outcomes.

## Ethical approval

This case study was reviewed by the local office of research governance and development and deemed exempt from ethical review on the basis that it is a case report—EX/2024/QGC/114867.

## Patient consent

Written informed consent has been provided by the patient in the case study to publish this case report.

## References

[1] Bergengren O., Pekala K.R., Matsoukas K. (2023). 2022 update on prostate cancer epidemiology and risk factors—a systematic review. Eur Urol.

[2] Siegel R.L., Miller K.D., Fuchs H.E., Jemal A. (2022). Cancer statistics, 2022. CA: Cancer J Clin.

[3] Posdzich P., Darr C., Hilser T. (2023). Metastatic prostate cancer—a review of current treatment options and promising new approaches. Cancers.

[4] Patel N., Mehawed G., Dunglison N. (2023). Uro-symphyseal fistula: a systematic review to inform a contemporary, evidence-based management framework. Urology.

[5] Garg G., Deliso M., Li S., Sharma P., Abdelbaki A., Gupta N. (2018). Prostatosymphyseal fistula after transurethral resection of the prostate (TURP), a rare and difficult to recognize complication. Urol Case Rep.

[6] Smeyers L., Borremans J., Van der Aa F., Herteleer M., Joniau S. (2024). The complex challenge of urosymphyseal fistula and pubic osteomyelitis in prostate cancer survivors. Eur Urol Open Sci.

[7] Patel K., Butt H., Patel S., Roux J., Bhagat A. (2022). Imaging findings of urosymphyseal fistulas. BJR|Case Rep.

[8] Takekawa K., Horiguchi A., Ojima K. (2023). Urosymphyseal fistula development following treatment for radiation-induced urethral stenosis in three patients with prostate cancer. IJU Case Rep.

[9] Wasserman P.L., Way A., Baig S., Gopireddy DR. (2020). MRI of myositis and other urgent muscle-related disorders. Emerg Radiol.

